# Multi-platform diagnostic strategy and biosecurity as basis of contagious agalactia control programs in endemic areas

**DOI:** 10.1186/s13620-025-00288-1

**Published:** 2025-01-18

**Authors:** Christian De la Fe, Ana Grau, Olga Minguez, Antonio Sánchez, Rosario Esquivel, Juan C. Corrales

**Affiliations:** 1 Departamento de Sanidad Animal, Facultad de Veterinaria, Universidad de Murcia, Campus de Espinardo s/n., Murcia, 30100 Spain; 2 Servicio de Sanidad Animal de la Dirección General de Producción Agrícola y Ganadera de la Consejería de Agricultura, Ganadería y Desarrollo Rural de la Junta de Castilla y León, León, Spain; 3 Dirección General de Salud Pública, Consejería de Sanidad de la Junta de Castilla y León, León, Spain

**Keywords:** Contagious agalactia, Small ruminants, Control program, *Mycoplasma*

## Abstract

**Background:**

Control strategies against contagious agalactia (CA), considered one of the most important diseases affecting small ruminants in countries surrounding the Mediterranean, are mainly based on traditional strategies considered suboptimal such as the use of inactivated vaccines and antibiotics. This manuscript analysed the efficacy of an alternative non-mandatory official control programme based on a multi-platform diagnostic panel and biosecurity developed and started in 185 herds placed in a contagious agalactia endemic area of Spain, using the data of 74,080 samples collected and analysed during a 4 years-period (2018–2021).

**Results:**

Globally, the combined analyses of bulk tank milk (BTM), ear or nasal swabs (in goats or sheep, respectively) and the serology to detect *Mycoplasma agalactiae* (*Ma*), allowed the detection of 40.54% of positive farms (*n* = 75), with *Ma* the species always detected in sheep (100%) and *Mycoplasma mycoides* subsp. *capri* (*Mmc*, 68,8%), *Ma* (29,3%) or both of them (1,9%) in goats. Taking into account productive aptitude and vaccination against CA, the use of BTM, ear or nasal swabs, and serology for herd classification demonstrated either a lack of concordance or only weak concordance. In herds that did not vaccinate, the classification of positives by male serology or swab detection showed moderate concordance. Vaccination against AC proved to be a protective factor against the occurrence of herds with bucks or rams testing positive.

**Conclusions:**

Since the different diagnostic techniques are not interchangeable, it is necessary to apply a multi-platform diagnostic panel for the accurate classification of herds. Based on official classification, strict biosecurity standards, including the prohibition of the entry of animals with unknown health status, allowed the completion of the CA control program.

## Background

Contagious agalactia (CA) is one of the most important diseases affecting small ruminants, especially at the Mediterranean countries [1–3] where financial losses are high and probably underestimated [4]. *Mycoplasma agalactiae* (*Ma*) is the main causal agent of the diseases in both sheep and goats, followed by *Mycoplasma mycoides* subsp. *capri* (*Mmc*), only in goats. *Mycoplasma capricolum* subsp. *capricolum* or *Mycoplasma putrefaciens* are also involved in CA but they are only sporadically isolated in goats [1, 5].

In affected areas, acute or hyperacute outbreaks are usually characterized by the classical clinical description of the syndrome (mastitis, arthritis or conjunctivitis), which are rarely described in the same animal or even at the same herd. Nevertheless, chronically infected herds are the dominant epizootic situation registered in many countries. In these herds, it´s common the presence of subclinical mastitis in a percentage of the animals, which, in some cases, evolve to clinical mastitis and mammary gland atrophy [2, 3]. For this reason, the presence of both apparent asymptomatic infected males and females with microscopic lessons is also frequent [6], being necessary a continuous sampling strategy to detect their presence, thus complicating diagnosis and control and promoting the disease dissemination among herds [7].

CA control strategies have been traditionally based on the use of inactivated vaccines and antibiotics [4], even considering the limitations that still both tools have [3]. Bacterins are not able to prevent the infection or even the appearance of clinical signs in vaccinated animals and they are considered suboptimal [8]. Despite it, they are widely used because sometimes, by non-demonstrated reasons, commercial and autogenous vaccines, used 3–4 times per year, can decrease the severity of clinical symptoms [4, 9].

Similarly, antibiotics have been used to decrease the clinical impact of the disease when a CA outbreak occurred. In these cases, the use of antibiotics such us quinolones, macrolides or tetracyclines joined to partial stamping out of more affected animals have probably been the only strategy to limit the clinical effects on the herd. Despite that, the bacterial cure of affected animals is not achieved, and the infection always persist [3, 6]. Moreover, antibiotic resistances have emerged in the last decades as one of the most important sanitary problems worldwide and several studies evidenced the presence of CA-causing mycoplasmas resistant strains against enrofloxacin, marbofloxacin or tylosine [10–14]. Thus, official programmes emerged [15] promoting a most coherent use of antibiotics and opening the way to limit the use of some antibiotics in livestock to preserve their use for humans, such as fluoroquinolones, limiting the therapeutic options available to control CA outbreaks.

Alternative control initiatives based on diagnosis and biosecurity to define the status of the herds are scarce and limited to some areas of Europe, with notable differences in notification systems, mandatory or voluntary participation, the biosecurity measures adopted, or the diagnostic strategies used [4, 16, 17]. Basically, these initiatives are designed to prevent the entry of mycoplasmas in CA free-areas or herds, although surveillance studies developed on large groups evidenced the complexity of the goal [5, 18]. In Spain, a novel non-mandatory official CA control programme based on a multi-platform diagnostic panel and the application of strict rules of biosecurity has been developed in Castilla y Leon -an endemic CA area with an important dairy sheep production- [19, 20]. In this area, the main health and hygiene management practices that affect milk quality have been identified [21] and the presence of mycoplasmas represents a serious threat to the viability of the sector. This strategy has been latter assumed at National level by the Spanish Ministry and is now open to every region or herd of the country that can apply [22]. This manuscript analysed the efficacy of this combined strategy to fight against CA in endemic areas analysing the results obtained after a 4 years-period of work (2018–2021).

## Methods

### CA control programme design

The programme, conceived as a non-mandatory action permanently open for new incorporations, was developed considering three basic aspects: (1) The movements are forbidden for participants during a period of 3 years, with the exception of herds with the same sanitary status, because the entry of animals is the main factor involved in CA-causing mycoplasmas outbreaks; (2) the herds are permanently monitored using different diagnosis techniques based on the livestock aptitude, and (3) the detection of asymptomatic carriers is very important, because of their high presence previously reported in chronically infected herds [23, 24].

On the basis of these premises, Table 1 summarized the key points of the multi-platform diagnostic panel used in each herd according to the productive aptitude, the vaccination status and the ruminant species studied. All the samples used, such as bulk tank milk (BTM) [16, 17], blood serum [25, 26] or the swabs collected from males [27, 28] has been successfully used to detect infected animals or herds, but to date never combined for this purpose. Obviously, serology was only used in non-vaccinated herds and bulk tank milk analyses were only conducted on dairy herds. Ear or nasal samples were used to detect the presence of carriers for goat bucks or rams, respectively, according to published information [28, 29].

**Table 1:** Diagnostic strategy in the contagious agalactia (CA) voluntary programme according to the productive aptitude and vaccination status of the ovine and caprine herds studied

Productive aptitude	Vaccination status	Cohort tested and/or sample	Chronology	Diagnostic testing
Milk	CA vaccination	Bulk tank milk	5 samples/year	PCR^2^
		Bucks or rams/swab^1^	Each 6 months	PCR^2^
	Non-CA vaccination	Bulk tank milk	5 samples/year	PCR^2^
		Bucks or rams/swab^1^	Each 6 months	PCR^2^
		All > 4-year animals/blood	Each 6 months	Serology
Meat	CA vaccination	Bucks or rams/swab^1^	Each 6 months	PCR^2^
	Non-CA vaccination	Bucks or rams/swab^1^	Each 6 months	PCR^2^
		All > 4-year animals/blood	Each 6 months	Serology

^1^Auricular or nasal swab for bucks or rams, respectively

^2^ RT-PCR for detection of *Mycoplasma agalactiae* in ovine and caprine samples and for *M. putrefaciens* and mycoides cluster in caprine samples

### Sample collection and diagnostic methods

Samples were always collected in the herds by official specific staff, refrigerated (4ºC), and immediately sent to the “Laboratorio Regional de Sanidad Animal” (León, Spain), where the diagnostic was conducted. For serological analyses, blood samples (*n* = 22,979) were collected from unvaccinated males and females higher than 4-years old placed in the herds (2018–2021). The presence of specific antibodies against *Ma* was then conducted by indirect ELISA (IDEXX *M. agalactiae* Screening Ab Test). BTM samples (*n* = 2,344), 2 ear swabs or nasal swabs samples (*n* = 46,926) were also taken when corresponded to detect the presence of CA-causing mycoplasmas. DNA extractions were conducted from samples using an automated extraction instrument (King Fisher Flex 96, Thermo Scientific), using the Mag Max Core nucleic acid purification kit following the manufactur´s instructions. Later, RT-PCR was conducted in 2 ul of extracted DNA using the kit VetMAX™ *M. agalactiae* & *M. mycoides* Kit https://www.thermofisher.com/order/catalog/product/TMYCAS50, in a termocycler Quantstudio 5 (Applied Biosystems), following the manufacturer´s recommendations. When necessary, the mycoplasma species involved in the samples yielding a positive result for *M. mycoides* cluster was confirmed using a primer pair to amplify the *fus*A gene [30]. These PCR products were finally sequenced (Universidad de Murcia, Murcia, Spain). Sequences were aligned using MEGA 6.0 software [31] and trimmed to the same size, providing fragments of 561 bp for phylogenetic analyses. DNA extracted from the reference strains of Ma (PG2, NCTC 10123) and Mmc (PG3, NCTC 10137) was included as positive control in these cases.

### Population studied

Details of the population studied are showed in Table 2. Analyses were conducted in 185 herds (154 ovine vs. 31 caprine), mainly of dairy aptitude (175 vs. 10), and comprising a population of approximately 178,000 small ruminants, mainly sheep (157,000 vs. 21,000), placed in a CA endemic area, as previously exposed [19, 20]. The breeds studied included Churra, Assafe or Lacaune (sheep) or Murciano-Granadina (goats) managed under intensive or extensive conditions (for dairy or meat aptitude, respectively).

**Table 2:** Productive aptitude and size of the herds studied

Species	Productive aptitude	Herds (*n*)	Total	Census	
				Means ± SD	Range
Ovine	Meat	9	5,024	558.22 ± 356.89	192-1,337
	Milk	145	152,007	1,048.32 ± 827.89	139-4,496
Caprine	Meat	1	44		
	Milk	30	21,772	725.73 ± 472.61	121-2,247
Total		185	178,847	966.74 ± 777.59	44 − 4,496

### Statistical analysis

Statistical analyses were run using the EpiInfo software (Epi Info™. Available online: https://www.cdc.gov/epiinfo/index.html) using ANOVA or Mann–Whitney/Wilcoxon Two-Sample Test (Kruskal–Wallis test for two groups) according to the inequality of population variances. The agreement test between the herd classification according to the diagnostic strategy was carried out by estimating the Cohen’s kappa coefficient at a 95% confidence interval, using the WinEpi program (http://www.winepi.net/). The criteria for result interpretation were based on Thrusfield [32]. The relationship between the vaccination status of the herd and the factors studied was carried out by estimating the Odds Ratio using the WinEpi program.

## Results

### Detection of CA-causing mycoplasmas in BTM and swabs

A total of 49,270 samples were analysed during the period studied to detect the presence of CA-causing mycoplasmas in BTM (*n* = 2,344 samples from 175 dairy herds) and swab samples collected from males (*n* = 46,926 samples from 10,648 animals). Table 3 summarized the results obtained. Globally, Ma was the mycoplasma species identified in all the samples collected in sheep (100%). The presence of *Ma* (29,3%), *Mmc* (68,8%) or mixed infections with both agents (1,9%) was detected in goats. *Mcc* or . *M.putrefaciens*  were not detected. A total of 2,344 BTM samples were collected during these 4 years, most of them, taken in ovine herds, in a proportion 5 vs. 1. Ma was the only species detected in BTM in both goat and sheep herds. Globally, 0.84% of the swab’s samples were positives, half of them for each ruminant species, thus representing a higher percentage of carriers in goats vs. sheep (3.71% vs. 0.48%). *Mmc *was the most detected mycoplasma in auricular swabs: *Mmc* (88.2%), *Ma* (9.4%) or mixed infections with both agents (2.4%), while only *Ma* was detected in nasal swabs taken from sheep (100%).

**Table 3:** Global samples and results obtained in the 185 herds ascribed at the voluntary programme

Cohort or sample tested	Ovine	Caprine	Total
Bulk tank milk, n	1,910	434	2,344
BTM positives, n (%)	108 (5.65%)	41 (9.54%)	149 (6.36%)
Buck/rams studied, n	9,418	1,230	10,648
Bucks/rams swab samples, n	41,584	5,342	46,926
Bucks/rams swab positive samples, n (%)	198 (0.48%)	198 (3.71%)	396 (0.84%)
Bucks/rams only swab positives animals, n (%)	30 (0.32%)	23 (1.87%)	53 (0.5%)
> 4-year Bucks/rams serology samples, n	1,552	279	1,831
> 4-year Bucks/rams serology positive samples, n (%)	75 (4.83%)	30 (10.75%)	105 (5.73%)
> 4-year Bucks/rams only serology positive animals, n	35	5	40
> 4-year Bucks/rams swab and serology positive animals, n	24	18	42
Bucks/rams total positive animals	89	46	135
> 4-year females studied*, n	16,415	6,564	22,979
> 4-year females’ serology positive animals, n (%)	1,081 (6.59%)	190 (2.89%)	1,271 (5.53%)

*Only in 61 non-vaccinated herds

### Serology

A total of 24,810 samples were analysed to detect the presence of antibodies against *Ma*. Most of the samples pertaining 4-years females placed in non-vaccinated herds but samples from 1,831 males were also collected. Table 3 summarized the results obtained. As we can see, in females, the overall percentage of sheep scored positives was higher than goats. Nevertheless, the 10% of goat bucks scored positives in comparison with rams (10.75 vs. 4.83), although this global percentage is probably affected by the high percentage of carriers detected in one of the caprine herds studied.

### CA status of the herds

The qualification of a herd as CA positive resulted from the combination of all the diagnostic strategies applied according to the type of herd (Table 1). Table 4 showed the classification of the herds according to the category and the group of animals or sample studied. Of the herds studied, 40.54% (75 out of 185) was classified as CA positive. Among the dairy herds (*n* = 175), CA-causing mycoplasmas were detected in 72 positive farms (41.14%), representing a 50% in caprine and 39.31% in ovine herds, respectively.

**Table 4:** Results of the classification of the herds according to the herd category and type of cohort or sample tested

Herd category^1^	Type of cohort or sample tested	Specie	Herds Positives^2^/Total in the category (%)	Positive herds by the type of cohort or sample tested and frequency of positive herds identified (%)
Dairy herds	Bulk tank milk		72/175 (41.14%)	37 (51.39%)
		Caprine	15/30 (50%)	6 (40%)
		Ovine	57/145 (39.31%)	31 (54.39%)
All herds	Bucks/rams^2^		75/185 (40.54%)	32 (42.67%)
		Caprine	15/31 (48.38%)	7 (46.67%)
		Ovine	60/154 (38.96%)	25 41.67)
All herds	Bucks/rams with swab positives animals		75/185 (40.54%)	22 (29.33%)
		Caprine	15/31 (48.38%)	6 (40%)
		Ovine	60/154 (38.96%)	16 (26.67%)
Non-vaccinated herds	> 4-year Bucks/rams with serology positive animals		35/61 (57.38%)	14 (40%)
		Caprine	8/19 (42.11%)	2 (25%)
		Ovine	27/42 (64.29%)	12 (44.44%)
Non-vaccinated herds	> 4-year Bucks/rams swab and serology positive animals		35/61 (57.38%)	13 (37.14%)
		Caprine	8/19 (42.11%)	2 (35%)
		Ovine	27/42 (64.29%)	11 (40.74)
Non-vaccinated herds	> 4-year females seropositives		35/61 (57.38%)	27 (77.14%)
		Caprine	8/19 (42.11%)	6 (75%)
		Ovine	27/42 (64.29%)	21 (77.78%)

^1^ Defined by the productive aptitude or the vaccination status

^2^ Classified combining all the diagnostic strategies defined for each type of herd

Considering the diagnosis sample used to classify the herds, the use of BTM allowed the detection of 51.39% (37/72) of the positive herds (40% in caprine and 54.39% in ovine herds). Detection of positive bucks/rams by serology and/or swabs, according to the vaccination status, identified a total of 42.67% (32/75) of the positive herds (46.67% in caprine and 41.67% in ovine herds), while the exclusive use of ear or nasal swabs identified 29.33% (22/75) of the positive herds (40% in caprine and 26.67% in ovine herds). Interestingly, serology of males identified 40% (14/35) of the positive herds (25% in caprine and 44.44% in ovine herds) in non-vaccinated herds, while the study of the blood collected from females qualified 77.14% (27/35) of the positive herds (75% in caprine and 77.78% in ovine herds) (Table 4).

Individually, Table 5 shows the mean number of positive samples/animals in the herds classified as CA positive according to the group of samples or animals studied. Herds positive to BTM samples (*n* = 37) presented a mean of 4.03 positive samples per herd (6.83 in caprine and 3.48 in ovine herds, *p* < 0.05), being the mean frequency of positive samples per herd 29% (47.53% in caprine and 25.41% in ovine herds, *p* < 0.05). In the herds with positive sires qualified by the combination of the different diagnostic strategies (*n* = 32), the mean number of positive sires per herd was 4.22, showing no significant differences between species. In the 14 herds that did not vaccinate and that presented positive serological males, the mean number of seropositive animals was 2.5, showing no significant differences between species. In the 27 herds that were not vaccinated and that presented seropositive females, the mean number of animals per herd was 47.07, showing no significant differences between species. The mean frequency of seropositive females per herd was 17.56%.

**Table 5:** Characteristics of the *Mycoplasma* spp. positive herds according to the type of sample and cohort studied

Mycoplasma spp. positive herds			Positive samples/animal by herd Means ± SD (Range)	Frequency (%) of positive samples/animal by herd Means ± SD (Range)
Type of cohort or sample tested	Specie	n*		
BTM positives		37	4.03 ± 2.81 (1–12)	29 ± 17.37 (5.88-75)
	Caprine	6	6.83^a^ ± 4.02 (1–12)	47.53^a^ ± 17.76 (25–75)
	Ovine	31	3.48^b^ ± 2.22 (1–10)	25.41^b^ ± 15.09 (5.88–61.54)
Bucks/rams positives		32	4.22 ± 5.93 (1–30)	Nd
	Caprine	7	6.57^a^ ± 10.49 (1–30)	
	Ovine	25	3.56^a^ ± 3.99 (1–18)	
Bucks/rams with swab positives animals		22	4.42 ± 5.78 (1–18)	
	Caprine	6	4.6^a^ ± 5.94 (1–15)	Nd
	Ovine	16	4.29^a^ ± 6.13 (1–18)	
> 4-year Bucks/rams with serology positive animals		14	2.5 ± 2 (1–8)	
	Caprine	2	2.5^a^ ± 0.71 (2–3)	Nd
	Ovine	12	2.5^a^ ± 2.14 (1–8)	
> 4-year Bucks/rams swab and serology positive animals		13	3.23 ± 3.88 (1–15)	
	Caprine	2	9 ± 8.49 (3–15)	Nd
	Ovine	11	2.19 ± 1.72 (1–6)	
> 4-year females seropositives		27	47.07 ± 111.18 (1-545)	17.56 ± 27.3 (1.12–100)
	Caprine	6	31.37^a^ ± 69.3 (1-173)	8.47^a^ ± 17.87 (0.14–44.82)
	Ovine	21	51.48^a^ ± 121.56 (1-545)	20.15^a^ ± 29.28 (0.12–100)

BTM: bulk tank milk

* At least one positive sample or animal by herd

^a, b^: means with different superscript between species differ significantly (*p* < 0.05) in the cohort tested or sample studied

Nd: not determined since the combination of serology samples and swabs studied in bucks/rams varies according to herd vaccination strategy

Table 6 shows the agreement test between the herd classification according to the diagnostic strategy used. In dairy herds without CA vaccination (*n* = 54), there was no agreement between the use of BTM and female serology or between BTM and the detection of positive males. In these herds, the concordance between the qualification of the herds by serology of the females and the status of the sires was weak (Kappa coefficient = 0.34). Likewise, in all dairy herds (*n* = 175), the use of BTM did not show concordance with the status of the sires. In herds that did not vaccinate, the classification of positives by male serology or swab detection showed moderate concordance (Kappa coefficient = 0.59).

**Table 6:** Agreement test between the herd classification in the CA voluntary program according to the diagnostic strategy

	Non-vaccinated dairy herds			All dairy herds	Male status^1^
	BTM/Female serology	BTM/Male status^2^	Female serology/Male status^2^	BTM/Male status^2^	Male serology/swab
Kappa coefficient	-0.09	0.12	0.34	0.09	0.59
CI for kappa se(0)	-0.28, 0.10	-0.12, 0.36	0.09–0.59	-0.06- 0.23	0.34–0.83
CI for kappa se(1)	-0.225, 0.05	-0.04, 0.28	0.16, 0.5	-0.02, 0.19	0.36–0.82
Observed agreement	50%	70.4%	68.5%	71.4%	86.9%
Expected agreement	54.1%	66.5%	52.5%	68.6%	68.2%
Observed agreement minus hazard	-4.12%	3.9%	16%	2.8%	18.7%
Maximum agreement not due to hazard	45.9%	33.5%	47.5%	31.4%	31.8%
Concordant values					
Negative herds	25	35	26	116	45
Positive herds	2	3	11	9	8
Discordant values	27	16	17	50	8
Total studied herds	54	54	54	175	61

BTM: bulk tank milk

^1^In non-vaccinated herds

^2^Classsified using swab and/or serology in > 4-year animals according to vaccination strategy of the herd

Table 7 shows the relationship between the vaccination of the herd and the species, the BTM results and the bucks/rams status. Vaccination was mostly used in sheep collectives. It is not associated with qualification on the basis of the BTM use. However, vaccination appears as a protective factor against the occurrence of flocks with positive sires; vaccinated flocks are 2.38 times less likely to have positive males.

**Table 7:** Relationship between the vaccination of the herd and the specie, the bulk tank milk (BTM) results and the bucks/ram’s status

		Herd vaccination		
		Yes	No	
Species	Caprine	12 (38.71%)	19 61.29%)	*P* < 0.001
	Ovine	112 (72.73%)	42 (27.27%)	
BTM	Positives	30 (81.08%)	7 (18.92%)	OR = 2.21 (0.9 < OR < 5.42)
	Negatives	91 (65.94%)	47 (34.06%)	
Bucks/Rams^1^	Positives	16 (50%)	16 (50%)	OR = 0.42 (0.19 < OR < 0.89)
	Negatives	108 (70.59%)	45 (29.41%)	

^1^In vaccinated herds, the diagnosis of bucks and rams was performed using PCR on ear and nasal swabs, respectively. In non-vaccinated herds, serology by ELISA was also conducted on animals older than four years

## Discussion

This manuscript analyses the suitability of a novel approach to fight against CA in endemic areas. Based on a continuous diagnosis using different techniques combined with the application of a strict policy of biosecurity, we used the information recovered in Castilla-Leon (Spain) during a 4-years period of application, to analyze its suitability (Tables 1, 2 and 3). By the number of samples analyzed, this study represents a good opportunity to test the efficacy of most of the diagnostic tools available to fight against the diseases when used and combined on a large scale. As it could be expected according to the data in the literature, results showed that only *Ma* was detected in sheep while both *Ma* and *Mmc* were detected in goat herds.

Technically, a first approach to the data obtained showed that all the samples and diagnostic methods used are valid to detect the presence of infected animals and especially, they are all necessary for the correct qualification of the herds. In effect, there are infected flocks classified exclusively by the presence of mycoplasmas in BTM, the presence of asymptomatic carriers or the detection of seropositive individuals, both in sheep and caprine herds (Tables 4 and 5). The intermittent excretion of mycoplasmas in chronically infected herds previously reported [6] is here confirmed, making it necessary to use different techniques to detect as many infected flocks as possible. In this regard, the obtained results demonstrate the lack of concordance among the diagnostic techniques studied (Table 6), indicating that they cannot be used interchangeably for the accurate classification of herds. This finding underscores the need for careful consideration when selecting diagnostic tools, as each technique provides distinct results that cannot be substituted for one another in ensuring the correct assessment of herd status.

The results obtained showed that BTM should be also used for control purposes, as a basic and economic tool for CA control in endemic areas, as previously proposed [16, 17, 19]. However, the intermittent excretion of mycoplasmas and the presence of a limited number of infected ones may affect the PCR results [17] and explain the presence of herds where the BTM is systematically negative, and yet the presence of asymptomatic carriers is evidenced in the collective of females or males (Table 4). The patterns of excretion of mycoplasmas in milk were not correlated with breeding factors (drying periods, batching stress, food stress, etc.), somatic cell counts [17], or the lactation stages [19], so several samples will be required over time. In this sense, even though at least 5 samples are necessary throughout lactation to detect the presence of infection in infected herds [16], the level of detection of the CA positive herds obtained (51.39%) using BTM show that a CA control program cannot be based solely on the use of this sample.

The use of the males as a sentinel to detect the infection is undoubtedly another major novelty of the programme. The sample size of this study, representing the higher number of ear or nasal swabs ever collected and analysed for CA diagnosis, as the author´s knowledge, seems to confirm its usefulness for CA control programmes [24, 27, 28, 33]. In the current study, analysis of nasal or auricular swabs detected a total of 89 and 46 CA-infected rams and goat bucks, respectively, thus representing an overall percentage close to 1% of carriers among those sampled. More importantly, it was the tool used to detect CA-causing mycoplasmas in 22 herds. These data confirmed the transcendental epizootic role of asymptomatic carriers in chronically infected areas, where their uncontrolled movement between the herds is highly related to the presence of CA clinical outbreaks [6]. By species, the higher percentage of carriers was detected in goats, thus confirming the importance of ear carriers on caprine farms. In these herds, *Mmc* was the most isolated species, confirming the presence of this mycoplasma in this type of sample [24, 33–35], an anatomical area of predilection where you can find these bacteria just a few weeks after an experimental infection [29].

In reference to the diagnostic use of serology, the presence of infected individuals repeatedly testing negative with this technique has been common in this work, as previously observed [35]. Several factors, such as the presence of surface antigenic variability mechanisms in bacteria like *Ma* or *Mmc*, including some of the surface lipoproteins associated with the host immune response; or the experimental demonstration of the blockage in antibody production generated by *Ma* a few weeks after the onset of infection, contribute to explain these findings [36]. However, the results obtained demonstrate the validity of serology to be used at herd level when herds are not vaccinated, allowing the analysis of a large number of animals at a relatively low cost [25, 37]. However, it seems important to select the type of test to be used [25, 26], and the type of animals against which it is used. In this case, ELISA was used only with the animals that had been on the herd for at least 4 years, avoiding its use on the youngest individuals, as well as being used on the males, which are in contact with all the individuals in each herd. Indeed, serology was only useful for detecting some of the rams or goat bucks in which the presence of *Ma* was detected.

Although it is not an objective of this work to address the specific situation of the infection in Castilla y León, the results obtained during these 4 years are consistent in evidencing the absence of mycoplasmas associated with contagious agalactia in 51% of the herds analyzed, which constitutes a very interesting percentage of animals when it comes to being able to count on herds free of the infection in future eradication strategies. We must not forget that this strategy is compatible with the use in both vaccinated and non-vaccinated herds or herds requiring timely use of antibiotics. By prohibiting the entry of animals of unknown health status, the risk of entry of infected individuals is reduced, a key factor for new disease outbreaks [38] and independent of the use of other control strategies such as vaccination [8]. In this sense, the percentage of sheep flocks that vaccinate (72,73%) was similar to the previous one recorded in the same area [21] and significantly higher compared to goats, which may be related to the presence of a single agent -*Ma*- and to the lower variability of the circulating strains in this ruminant species [39, 40]. This could explain why vaccination appears as a protective factor against the appearance of herds with positive sires.

The results obtained indicate that the surveillance of CA requires combined diagnostic structures, which facilitate the design of control programs and reflect the situation of CA in endemic areas. In this regard, the lack of official epizootic data in member states has been proposed as the reason for the exclusion of CA from the animal health legislation of the European Union, perpetuating CA as a neglected disease and contributing to its under-reporting [41].

Despite the advantages provided by means of the use of various diagnostic strategies in CA control programs, one of the primary limitations lies in the economic cost of this approach. The financial support from governmental administrations to cover both the official collection and transport of samples as well as the costs of analyses is necessary. Furthermore, the availability of accredited laboratories is essential for the large-scale application of these techniques. Both factors can hinder the development of control programs, particularly in countries where other animal health priorities exist.

## Conclusions

Since the different diagnostic techniques are not interchangeable, it is necessary to apply a multi-platform diagnostic to assess the health status of the herds. Based on official classification, strict biosecurity standards -together with the prohibition of the entry of animals with unknown health status-, allowed the completion of the CA control program.

## Data Availability

No datasets were generated or analysed during the current study.

## Abbreviations

BTM: Bulk tank milk
CA: Contagious agalactia
Ma: *Mycoplasma agalactiae*
Mmc: *Mycoplasma mycoides* subsp. *capri*
